# Storage of Extended Boar Semen at 5 °C Inhibits Growth of Multi-Drug Resistant *Serratia marcescens* and *Klebsiella oxytoca* while Maintaining High Sperm Quality

**DOI:** 10.3390/antibiotics12050857

**Published:** 2023-05-05

**Authors:** Isabel Katharina Maaßen, Anne-Marie Luther, Jutta Verspohl, Dagmar Waberski

**Affiliations:** 1 Unit for Reproductive Medicine, Clinic for Pigs and Small Ruminants, University of Veterinary Medicine Hannover, Foundation, Bünteweg 15, D-30559 Hannover, Germany; isabel.katharina.maassen@tiho-hannover.de (I.K.M.); anne-marie.luther@tiho-hannover.de (A.-M.L.); 2 Institute for Microbiology, University of Veterinary Medicine Hannover, Foundation, Bischofsholer Damm 15, D-30173 Hannover, Germany; jutta.verspohl@tiho-hannover.de

**Keywords:** *Serratia marcescens*, *Klebsiella oxytoca*, boar semen, antibiotic resistance, semen preservation

## Abstract

Multi-drug antibiotic resistance of *Serratia (S.) marcescens* and *Klebsiella (K.) oxytoca* in boar semen is an emerging threat to pig reproduction and the environment. The aim of this study is to examine the efficiency of a novel hypothermic preservation method to inhibit the growth of these bacterial species in extended boar semen and to maintain the sperm quality. The semen samples extended in an antibiotic-free Androstar Premium extender were spiked with ~10^2^ CFU/mL of *S. marcescens* or *K.oxytoca*. Storage at 5 °C for 144 h inhibited the growth of both bacterial species and maintained the sperm quality, whereas bacterial counts increased to more than 10^10^ CFU/mL in the 17 °C samples used as positive controls. This was accompanied by an increase in the sperm agglutination and the loss of motility and membrane integrity. We conclude that hypothermic storage is a promising tool to combat resistant bacteria in boar semen and to contribute to the One Health approach.

## 1. Introduction

Artificial insemination (AI) with liquid-preserved semen is the most used biotechnology in pig reproduction worldwide [1]. Due to the high chilling sensitivity of boar spermatozoa [2], porcine semen is commonly stored between 16 and 18 °C. The relatively high storage temperature poses the risk of bacterial growth, thus enforcing the use of antibiotics in semen extenders. The continuous use of antibiotics, together with disinfectants in semen collection centers, has favored the generation and spread of multi-drug resistances [3]. Most of the bacteria usually occurring in the raw semen are commensal [4], belonging to the *Enterobacter*, and neither impact the sow’s health nor the sperm quality if their amount is below 10^7^ CFU/mL [5,6,7,8]. However, the contamination of extended semen with the opportunistic pathogens *Serratia marcescens* and *Klebsiella oxytoca* were identified as bacteria of high concern due to their fast growth in extended semen, together with a high spermicidal effect and multi-drug resistance [9,10,11,12]. These Gram-negative bacterial species, like many other bacterial species identified in preserved boar semen, may either originate from the animal or may enter the semen from the environment in the stable or laboratory [5,13,14]. *Serratia marcescens* and *K. oxytoca* are well known as nosocomial bacteria, typically acquiring resistances in locations with high exposure to antibiotics and disinfectants [15,16], such as in hospitals and laboratories of AI centers. The threat of bacterial contamination has increased the overuse of antibiotics in semen extenders, resulting in the loss of efficient antimicrobial control. Notably, the high rate of semen backflow from the sow’s reproductive tract after insemination [17] poses the risk of antibiotics and resistant bacteria entering the environment and, from there, the human food chain. These situations have stimulated intense research for alternatives to antibiotics in boar semen extenders [3,18], of which, to date, none have become established in the AI industry, either due to the lack of broad-spectrum efficiency, sperm toxicity, or high time and cost intensity. Recently, hypothermic storage of boar semen was proposed as an innovative preservation concept to keep bacteria below spermicidal levels [19]. Antibiotic-free semen storage at 5 °C proved to be efficient for inhibiting the growth of commensal bacteria naturally occurring in boar semen, and to maintain the high sperm quality and fertility in vivo [19,20,21]. Whether the hypothermic semen storage is also effective against multi-drug resistant bacteria remains to be shown. Beyond this background, this study aims to examine whether semen storage at 5 °C efficiently inhibits the growth of multi-drug resistant *S. marcescens* and *K. oxytoca* to levels that do not affect the sperm quality.

## 2. Results

**Figure 1:**
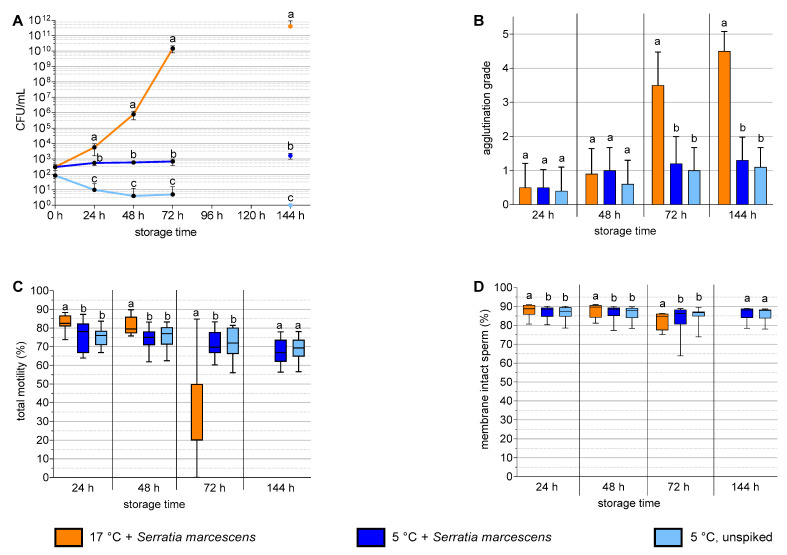
Bacterial growth (**A**), sperm agglutination (**B**), sperm motility (**C**), and sperm membrane integrity (**D**) in boar semen spiked with *Serratia marcescens* and stored at 5 °C or 17 °C in antibiotic-free Androstar Premium extender. At 144 h storage at 17 °C, motility and membrane integrity were not analyzed due to the high sperm agglutination. Experiment 1, *n* = 10 semen samples from 8 boars. a–c: Different lowercase letters indicate differences at a given storage time point (*p* < 0.05).

The results of Experiment 1 with *S. marcescens* are shown in Figure 1. The spiked semen samples stored at 17 °C showed exponential growth of *S. marcescens* in pure cultures to >10^11^ CFU/mL, whereas storage at 5 °C inhibited the bacterial count at ~10^3^ CFU/mL (Figure 1A). In the un-spiked samples stored at 5 °C, the bacterial load decreased below <10^1^ CFU/mL within 48 h (*p* < 0.05). At 24 h and 48 h, the sperm motility and membrane integrity were higher in the 17 °C samples compared to the 5 °C samples. At 72 h, the 17 °C samples showed an increase in the sperm agglutination (Figure 1B) and a decrease in the sperm motility (Figure 1C) and membrane integrity (Figure 1D). After 72 h storage, the sperm motility and membrane integrity were higher in the 5 °C samples compared to the 17 °C samples. At 144 h, the samples stored at 17 °C were not analyzed for motility and membrane integrity due to the high sperm agglutination. At all the time points, the sperm quality parameters did not differ between the 5 °C samples with and without added *S. marcescens*.

**Figure 2:**
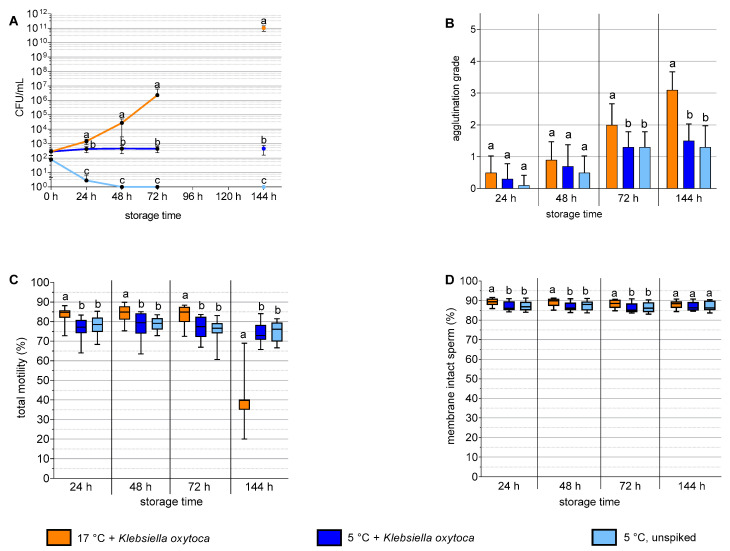
Bacterial growth (**A**), sperm agglutination (**B**), sperm motility (**C**), and sperm membrane integrity (**D**) in boar semen spiked with *Klebsiella oxytoca* and stored at 5 °C or 17 °C in antibiotic-free Androstar Premium extender. Experiment 2, *n* = 10 semen samples from 9 boars. a–c: Different lowercase letters indicate differences at a given storage time point (*p* < 0.05).

The results of Experiment 2 with *K. oxytoca* are shown in Figure 2. The spiked semen samples stored at 17 °C showed almost linear growth of *K. oxytoca* in pure cultures to ~10^11^ CFU/mL at 144 h, whereas storage at 5 °C inhibited the bacterial count to the initial spiking dose (Figure 2A). In the un-spiked samples stored at 5 °C, the bacterial load decreased below the detection limit (i.e., <10^1^ CFU/mL) within 48 h (*p* < 0.05). At 24 h, 48 h, and 72 h, the sperm motility and membrane integrity were higher in the 17 °C samples compared to the 5 °C samples. At 72 h and 144 h, the spiked 17 °C samples showed higher sperm agglutination compared to both types of 5 °C samples (Figure 2B). At 144 h, the sperm motility (Figure 2C) was higher in the 5 °C samples compared to the 17 °C samples, whereas the percentage of membrane-intact spermatozoa (Figure 2D) was at the same high level (greater than 85%) in all three sample types. At all the time points, the sperm quality parameters did not differ between the 5 °C samples with and without added *K. oxytoca*.

## 3. Discussion

The present study demonstrates that the preservation of extended boar semen at 5 °C efficiently inhibits the growth of multi-drug resistant *S. marcescens* and *K. oxytoca* during long-term storage. These data add to our previous observation that the growth of the two multi-drug bacterial species was inhibited at 5 °C compared to 17 °C in the semen-free extender medium [11]. In the present study, the inhibitory effect was shown using semen portions in the same way they would be used for insemination, i.e., in the presence of spermatozoa and seminal plasma. The latter is rich in proteins, lipids, and electrolytes [22], which provide nutrients for bacteria and thus could reduce the inhibitory effect of hypothermic semen storage. Here, by mimicking the contamination of semen portions with relatively high counts of two multi-drug resistant bacterial species isolated from boar semen, we showed that semen storage at 5 °C effectively kept the bacterial load far below spermicidal levels. By comparison of the bacterial growth at 5 °C storage in semen-free BTS (manufactured as a short-term extender) and the Androstar Premium (manufactured as a long-term extender), we previously demonstrated that the antimicrobial effect against *S. marcescens* and *K. oxytoca* is primarily caused by the low storage temperature and, to a lesser extent, the extender medium [11]. It is to note that the BTS extender does not protect the sperm against chilling injury [19], and therefore, is not applicable for 5 °C semen storage.

The thresholds for sperm damage were reported at >10^7^/mL for *S. marcescens* and >10^8^/mL for *K. oxytoca* [11]. In the present study, these levels were reached after 72 h of storage in the spiked 17 °C samples but not in the 5 °C samples. Similar to the observations with *Escherichia coli* [5] and *Enterobacter cloacae* [23], the sperm damage induced by *S. marcescens* and *K. oxytoca* was expressed at a bacteria/sperm ratio higher than 1:1.

Based on the studies with *E. coli* and *K. pneumonia* in human semen, bacterial adhesion to the sperm surface is regarded as a key event leading to increased sperm agglutination [24,25]. Similar to these observations, the sperm adherence of *S. marcescens* could be mediated by mannose-binding adhesion molecules detected on the pili and fimbria of this bacterial species [26,27] among further mechanisms, e.g., extracellular bacterial secretions or molecular aggregates as reported for *E. coli* [28]. The present study indicates that regardless of the cause for increased agglutination and the loss of sperm motility and membrane integrity, the sperm damaging effect is clearly related to the bacterial concentration, but not to the bacterial exposure time. This becomes evident in the spiked 5 °C samples, showing that the long-term exposition (144 h) to moderate counts of both *S. marcescens* and *K. oxytoca* did not induce a decline in the sperm quality. Confirming previous observations in the un-spiked 5 °C samples [19,20,21], the growth of commensal bacteria was well controlled below the detectable limits, but the loss of the sperm quality due to the chilling sensitivity of boar spermatozoa [2] is a challenge. Here, we used a recently established preservation protocol [19,29], considering that the type of extender medium and the slow controlled cooling are essential for high in vitro performance and fertility in vivo. Similar to the previous reports [19,20,21], there was a small decrease (less than 10 percent points) in the motility and membrane integrity in the 5 °C samples compared to the 17 °C samples with low bacterial counts in the first 48 h of storage. Notably, despite this initial chilling-associated loss of the sperm quality, the viable (i.e., membrane-intact) sperm population kept its functional integrity including its capacitation ability and mitochondrial activity during long-term storage [21]. This suggests that an impact on fertility is unlikely as long as sufficient numbers of viable spermatozoa are present, which has been previously confirmed by high fertility results in insemination trials under field conditions [19,21].

In conclusion, this study shows that the antibiotic-free storage of boar semen at 5 °C is effective not only against commensal bacteria naturally occurring in the raw semen, but also toward two multi-drug resistant bacterial species of highest concern in pig insemination. In the case of antibiotic ineffectiveness, currently, this seems to be the only option for an instant application in AI practice until the source of contamination is eliminated. When used routinely, antibiotic-free hypothermic semen storage presents a pathway toward sustainable pig reproduction in the sense of the One Health approach.

## 4. Materials and Methods

### 4.1. Semen Processing and Bacterial Inoculation

Semen was collected routinely once a week by trained personnel from nine mature, healthy boars (1 to 5 years of age) housed at the Unit for Reproductive Medicine, University of Veterinary Medicine Hannover, Hannover, Germany and treated in accordance with the European Commission Directive for Pig Welfare. After discarding the bulbourethral secretion, semen was extended to 20 × 10^6^ spermatozoa/mL in a final volume of 100 mL with the commercial antibiotic-free semen extender Androstar Premium (Minitüb GmbH, Tiefenbach, Germany). Extended semen portions were spiked with ~10^2^ CFU/mL *S. marcescens* (Experiment 1, *n* = 10 semen samples from 8 boars), or *K. oxytoca* (Experiment 2, *n* = 10 semen samples from 9 boars) isolated from commercial semen portions received from AI centers on the occasion of an annual semen quality control program conducted by our reference laboratory. Bacteria were cultured on Columbia agar with sheep blood (Oxoid Deutschland GmbH, Wesel, Germany) for 24 h at 37 °C before inoculation. Bacterial colonies were added to 2 mL extender medium and bacterial concentrations were adjusted after density photometry (SDM5, Minitüb, Tiefenbach, Germany). Immediately after inoculation (0 h), the bacterial count in the extended semen was determined. The bacterial species were identified with MALDI-TOF MS (microFlex LT, Bruker Daltonic, Bremen, Germany) and software Biotyper (Bruker Daltonic) before inoculation and at the end of semen storage. The Minimal Inhibitory Concentrations (MIC) for two common antibiotics in semen extenders, i.e., Gentamicin and Ampicillin, were evaluated by the microdilution method in the Institute for Microbiology at the University of Veterinary Medicine Hannover. For *S. marcescens*, MIC values for Gentamicin were ≥16 µg/mL, and for Ampicillin, 8 µg/mL. For *K. oxytoca*, MIC values for Gentamicin were ≥ 32 µg/mL, and for Ampicillin, ≥32µg/mL. Both bacterial species were considered as multi-drug resistant based on the susceptibility test with 16 different antibiotics [11]. Spiked semen portions were either stored at 17 °C (positive control) or slowly cooled to 5 °C following a previously established cooling protocol for the hypothermic storage of boar semen [29]. Additional semen portions remained un-spiked and were stored at 5 °C after slow cooling (negative control). All samples were stored for 144 h in the dark.

### 4.2. Bacterial Count

Bacterial counts were determined from 10-fold serial dilutions in PBS ranging from 10^−1^ to 10^−10^ and were plated in volumes of 100 µL on Columbia agar with sheep blood. After incubation for 24 h at 37 °C under aerobic conditions, bacterial colonies were counted, and the total bacterial numbers were calculated. Bacterial counts were expressed as colony-forming units per milliliter (CFU/mL).

### 4.3. Spermatology

Semen was examined at 24 h, 48 h, 72 h, and 144 h of storage. Sperm agglutinations were assessed with phase contrast microscopy (Carl Zeiss Microscopy GmbH, Jena, Germany) at 200× magnification. At least three different fields were examined, and the degree of agglutination was scored between 0 and 5 according to the estimated percentage of agglutinated spermatozoa as follows: 0 = 0 to 5%, 1 = less than 20%, 2 = 20 to 40%, 3 = 40 to 60%, 4 = 60 to 80%, 5 = 80 to 100%.

Sperm motility was measured as the total number of motile spermatozoa with the computer-assisted semen analysis (CASA) system AndroVision^®^ (Version 1.2, Minitüb GmbH, Tiefenbach, Germany). Subsamples were prewarmed at 38 °C for 30 min in a water bath under air and then filled in a 20 µL Leja chamber (Leja Products B.V., Nieuw Vennep, The Netherlands). At least 500 sperm were recorded with a frame rate of 30 pictures per 0.5 s. Motile spermatozoa were identified when their curved-line velocity was >24 µm/s and their amplitude of lateral head displacement was >1 µm. Sperm membrane integrity was assessed by flow cytometry using the Cyto Flex flow cytometer (Beckman Coulter GmbH, Krefeld, Germany) and the Cyt Expert 2.4. software (Beckman Coulter GmbH). Semen samples were stained in final concentrations with 1.3 µmol/L Hoechst 33342, 1.5 µmol/L propidium iodide (PI), and 2 µmol/L fluorescein conjugated peanut agglutinin (FITC-PNA). Fluorescence signals were detected in 10,000 events on the detectors FL-1 (450/45 nm BP), FL-2 (525/40 nm BP), and FL-3 (610/20 nm BP). Spermatozoa with intact plasma membranes and acrosomes were identified by a positive Hoechst stain and negative stainings for PI and FITC-PNA.

### 4.4. Statistical Analysis

Data analysis was performed with IBM SPSS Statistics Professional (SPSS Inc., IBM, Armonk, NY, USA). The normal distribution of data was checked with the Shapiro–Wilk Test. Data were then analyzed with the Friedman Test (XLSX). Pairwise comparisons were performed with the Wilcoxon Test and corrected with Holm Bonferroni. Values were considered as statistically significantly different when *p* < 0.05. Data are presented as mean and standard deviation (SD).

## Data Availability

The data presented in this study are available upon request from the corresponding author.
